# Met-Independent Hepatocyte Growth Factor-mediated regulation of cell adhesion in human prostate cancer cells

**DOI:** 10.1186/1471-2407-6-197

**Published:** 2006-07-25

**Authors:** Amanda Tate, Shuji Isotani, Michael J Bradley, Robert A Sikes, Rodney Davis, Leland WK Chung, Magnus Edlund

**Affiliations:** 1 Department of Urology and Winship Cancer Institute, Emory University School of Medicine, Atlanta, GA, USA; 2 Department of Biological Sciences, University of Delaware, Newark, DE, USA; 3 Department of Urology, Tulane University Health Sciences Center, New Orleans, LA, USA

## Abstract

**Background:**

Prostate cancer cells communicate reciprocally with the stromal cells surrounding them, inside the prostate, and after metastasis, within the bone. Each tissue secretes factors for interpretation by the other. One stromally-derived factor, Hepatocyte Growth Factor (HGF), was found twenty years ago to regulate invasion and growth of carcinoma cells. Working with the LNCaP prostate cancer progression model, we found that these cells could respond to HGF stimulation, even in the absence of Met, the only known HGF receptor. The new HGF binding partner we find on the cell surface may help to clarify conflicts in the past literature about Met expression and HGF response in cancer cells.

**Methods:**

We searched for Met or any HGF binding partner on the cells of the PC3 and LNCaP prostate cancer cell models, using HGF immobilized on agarose beads. By using mass spectrometry analyses and sequencing we have identified nucleolin protein as a novel HGF binding partner. Antibodies against nucleolin (or HGF) were able to ameliorate the stimulatory effects of HGF on met-negative prostate cancer cells. Western blots, RT-PCR, and immunohistochemistry were used to assess nucleolin levels during prostate cancer progression in both LNCaP and PC3 models.

**Results:**

We have identified HGF as a major signaling component of prostate stromal-conditioned media (SCM) and have implicated the protein nucleolin in HGF signal reception by the LNCaP model prostate cancer cells. Antibodies that silence either HGF (in SCM) or nucleolin (on the cell surfaces) eliminate the adhesion-stimulatory effects of the SCM. Likewise, addition of purified HGF to control media mimics the action of SCM. C4-2, an LNCaP lineage-derived, androgen-independent human prostate cancer cell line, responds to HGF in a concentration-dependent manner by increasing its adhesion and reducing its migration on laminin substratum. These HGF effects are not due to shifts in the expression levels of laminin-binding integrins, nor can they be linked to expression of the known HGF receptor Met, as neither LNCaP nor clonally-derived C4-2 sub-line contain any detectable Met protein. Even in the absence of Met, small GTPases are activated, linking HGF stimulation to membrane protrusion and integrin activation. Membrane-localized nucelolin levels increase during cancer progression, as modeled by both the PC3 and LNCaP prostate cancer progression cell lines.

**Conclusion:**

We propose that cell surface localized nucleolin protein may function in these cells as a novel HGF receptor. Membrane localized nucleolin binds heparin-bound growth factors (including HGF) and appears upregulated during prostate cancer progression. Antibodies against nucleolin are able to ameliorate the stimulatory effects of HGF on met-negative prostate cancer cells. HGF-nucleolin interactions could be partially responsible for the complexity of HGF responses and met expression reported in the literature.

## Background

In the prostate, cell-matrix adhesion, cell motility and invasive behaviors are regulated by an interplay of signals between the epithelial cells and surrounding stromal cells [1-6]. Signal reciprocity allows prostate stromal fibroblasts to control epithelial cell proliferation [7], while epithelial cells control such processes as stromal smooth muscle maturation [8]. When signal reception or intercellular signal interpretation alter adhesion-based behaviors, tumor formation and cancer progression can result. Cancer cells are known to optimize their stromal growth environments [6,9,10]. Indeed, the list of factors involved in bi-directional epithelial-stromal cell interactions is long, with representatives from many growth factor families, and includes Hepatocyte Growth Factor (HGF) [11], a subject of this study.

HGF regulates cell behaviors in organ development, tissue regeneration and cancer [12-15]. HGF's source, reception by, and effects on prostate cancer cells are discussed in many review articles, as is the Met protein, the only known cell surface receptor for HGF [16-21]. Once secreted, it is likely that most HGF is immobilized within the extracellular matrix of the stromal cells by binding heparan sulfate proteoglycans [22-26]. HGF encounters the Met receptor in the basal cells of the prostatic ducts and acini, and in low numbers on the luminal cells of the prostatic ducts and stromal smooth muscle cells [27-29]. During puberty, developing branches within the prostate show high concentrations of Met in ductal tips and respond to stromal stimulation [30,31], a hallmark for HGF/Met-mediated activity. Met signaling is also critical for ductal system formation in kidney, mammary gland, liver, pancreas and lung [32-35]. High levels of Met expression correlate with increased cell movement, and indeed metastasis is linked to the uncontrolled branching seen at earlier stages of prostate disease [36,37]. Furthermore, dysfunctional and high Met expression is found in a variety of human cancers [38,21,43] and correlates with some metastasis in animals [44,45].

Met expression levels during cancer progression remain somewhat confusing, and conflicting reports are common in the published literature. Met expression does appear to increase during prostate disease progression, but the correlation of Met expression with Gleason grade has been tenuous. Approximately 50% of localized cancers (and even more metastatic cancers) express Met [28,29,43,46]. In one study, Met elevations were found in 84% of localized prostate cancers [29], but Humphry et al. [28] reported that 45% of 108 cases show no correlation between disease progression and Met expression; further, the receptors in this study were localized by staining to both the cell surface and the cytoplasm. There are two other reports [43,46] of a clear increase in Met expression correlating with higher grades of adenocarcinomas (with metastases expressing more Met in bone than lymph node [29]), but no correlation between Met expression and disease progression, in a 5-year follow up period [43]. Not only are Met expression profiles not consistently linked to disease outcome [21,42,43], but Met expression is also confounding in the commonly-studied *in vitro *model systems. Met expression is higher in some metastatic prostate cancer samples compared to less-progressed cells [47-49]; for example, met RNA and protein levels are elevated in the androgen-independent cell lines DU145, PC3 and PC3M, compared to androgen-dependent LNCaP cells [27,28,43,50-54]. But, this correlation does not hold within the LNCaP-derived cell lines themselves, since neither parental LNCaP nor its lineage-derived, androgen-independent variant C4-2 actually express the HGF receptor Met. Thus, although both HGF and Met are arguably very important for prostate cancer progression, the details of their functions remain far from clear.

Further complicating Met/HGF correlations and prostate cancer models is the fact that high Met expression levels do not always invoke concentration-dependent responses to HGF treatment. For example, high-Met-expressing DU145 prostate cancer cells showed concentration-dependent responses to HGF, with increased cell motility in both scatter and invasion assays, whereas PC3 cells (with equally high levels of Met expression) did not respond under the same conditions [28]. These, and other contradictory reports of anti-apoptotic and pro-apoptotic responses to HGF treatment, have led some investigators to suggest that the lack of downstream signals explains differences between cell types [55], or that these differences may be due to isoform variants of HGF and Met themselves, or further that signaling pathway intermediates (such as PI3-kinase/Akt) may become saturated by extra-cellular matrix adhesion [56-63] and can not further be phosphorylated. We report here that cell adhesion to extra-cellular matrix does appear to play a role in cell spreading and migration response to HGF, as PC3 cells do respond to HGF treatment under our serum containing and starved growth conditions, but only when plated on laminin substrata. We and others, have been unable to detect any Met expression in LNCaP and C4-2 cells at either the protein or RNA levels (Figure 4; [21,43]), and yet we find a clear concentration-dependent response to HGF stimulation in these cells.

HGF likely acts through multiple isoforms, receptors and/or signaling cascades to bring about a variety of cell responses. Also called Scatter Factor (SF), HGF stimulates motility in both endothelial and metastatic epithelial cancers [53,55,64,65], similar to the invasion-promoting factor plasminogen [19,66,67]. Not surprisingly, HGF levels affect the function of prostate integrins [53,55], molecules involved in cell adhesion and motility. In this study, we have focused upon HGF's regulation of cell adhesive behaviors in a collection of human prostate cancer cell lines, including cell lines that do not express the Met receptor for HGF, but nonetheless exhibit distinct, concentration-dependent responses to the growth factor and to stromal-conditioned media (SCM). We previously reported that SCM increased cell spreading in the metastatic prostate cancer cell line C4-2, while having little effect on attachment of the lineage-related, non-metastatic LNCaP cell line [68]. We have now extended this work, further identifying HGF as responsible for the effects of SCM and describing HGF dose-dependent effects on the adhesive behaviors of these cell lines. In addition, we have especially searched for the responsible HGF receptors in these cells, as we and others have found both cell lines to lack the met protein, the one known HGF receptor ([43] and this study). Here, we introduce the protein nucleolin as a novel HGF binding partner in prostate cancer cells. Nucleolin, an abundant nuclear protein [69,70], is also found on the cell surface, where it has been shown to interact with heparin-bound growth factors [71-73], and where it functions as a cell surface receptor and a shuttle protein for nuclear import [72,74-76]. Significantly, nucleolin is also currently the focus of a phase II clinical trial as a cancer therapy target [77,78].

## Methods

### Cell culture and materials

Prostate epithelial and stromal cell lines were maintained in T-media with 5% fetal bovine serum, at 37°C with 5% CO_2_. Primary cultures of prostate stromal cells were derived from the tissue surrounding prostatic adenocarcinomas, as described by Ozen *et al*. [79]. Conditioned media were prepared by adding fresh media without serum when cells reached 80% confluence and removing it 48 h later. For signaling assays, cells were starved in RPMI-1640 phenol red-free medium (Life Technologies, Inc.) un-supplemented with serum. Laminin-1 (a kind gift of Roy Ogle at the University of Virginia) was purified from Engelbreth-Holme-Swarm (EHS) tumors according to the method of Davis et al., based on the protocol of Kleinman et al. [80,81]. Hepatocyte Growth Factor (HGF) and all other chemicals were purchased from Sigma (St. Louis, MO). Anti-FAK and HGF antibodies were from Sigma (St. Louis, MO) and Santa Cruz Biotechnology (Santa Cruz, CA). Antibodies to nucleolin were purchased from Santa Cruz Biotechnology or received as a gift from Dr. Deng at Pittsburgh Medical Center. Met antibodies, as well as cdc-42 and Rac immuno-precipitation reagent were purchased from both UpState Biotechnology (Lake Placid, NY) and Transduction Laboratories (Lexington, KY). Phospho-tyrosine antibodies were from Transduction Laboratories (Lexington, KY). Phospho-Akt (Ser 473) antibody and Akt antibody were purchased from Cell Signaling Technology Inc. (Beverly, MA). Anti-β actin antibody was from Abcam Inc. (Cambridge, MA). All secondary-conjugated antibodies were from Jackson Immunochemicals (West Grove, PA).

### Semi-quantitative reverse transcription PCR

Total cellular RNA was isolated with RNA-STAT (BioTec-X, Houston, TX). 5 μg of RNA was reverse transcribed using the OmniScript RT Kit (Qiagen, Inc., Valencia, CA). The primers used for met amplification were as follow: F-met 5'-GGTTGCTGATTTTGGTCAT-3' and B-met 5'-TTCGGGTTGTGGAGTCTT-3'.

#### Immunoprecipitation

Cells were allowed to grow to 80% confluency and then serum starved for 48 hours. Plates were rinsed twice in ice-cold phosphate buffered saline (PBS) and solubilized in lysis buffer (1% NP-40, 50 mM Tris-HCl, 150 mM NaCl, 2 mM EDTA, 50 mg/ml leupeptin, 0.5% aprotinin, 1 mM sodium orthovanadate, 1 mM PMSF). Insoluble material was removed by centrifugation for 30 min at 10,000 × g at 4°C. Protein concentration was determined by BRC assay (BioRad, Hercules, CA). 1–2 mg of protein was used for each immunoprecipitation condition. Antibodies were incubated with the cell lysate for 2 hrs at 4°C, and an additional 30 min with Protein A/G-sepharose beads (Sigma). The beads were washed three times with lysis buffer and resuspended in SDS-PAGE loading buffer. Samples were resolved on gradient (4–12%) or 10% polyacrylamide gels (Novex) and electro-blotted. After transfer, the filters were blocked in BSA (5%) overnight at 4°C. Filters were incubated with primary antibodies for 1 hr at room temperature. Membranes were then probed for 1 hr with peroxidase-conjugated secondary antibody (diluted 1:5000; Jackson Immunoresearch Labs, Bar Harbor, ME) and the proteins were detected with Enhanced Chemi-Luminescence (ECL)(Amersham Biosciences, Little Chalfont, England).

### Substrate adhesion and growth assay

Attachment assays were performed as previously described in Edlund et al., and Vafa et al., [68,82]. Cell lines were grown to confluence, trypsinized, and re-plated (1:8) on tissue culture dishes, where they were allowed to grow for another two days before being lifted after a brief treatment with 10 mM EDTA, 20 mM Hepes buffer in T-media. After neutralizing the EDTA with CaCl2 and MgSO4, the cells were washed with T-media containing 0.1% BSA. Cells were placed on laminin1-coated dishes, allowed to adhere for 30 to 90 min with or without addition of either HGF or function blocking integrin antibodies, and then fixed in para-formaldehyde (3.8%). The percentage of spread cells was scored for each cell line, based on cell membrane protrusion (lamellipodia and/or filopodia), and all values were normalized to control cells treated identically except for being subjected to conditioned media or growth factors. This normalization step was necessary because of the differences in speed of attachment between the cell lines. Cell growth was quantified using MTT [83,84].

### ELISA detection of HGF

ELISAs were performed as suggested in Pharmigen Research Products Catalog, 1999. Briefly, rabbit anti-human HGF antibodies were diluted to a concentration of 1 μg/ml in 0.1 M Na_2_HPO_4 _and 0.1 M NaH_2_PO, pH 9.0. Wells of a 96 well ELISA plate (Costar) were filled with 50 μl, sealed with parafilm, and incubated overnight at 4°C. Plates were then brought to room temperature and antibodies captured and removed. 200 μl of blocking buffer (10% FBS in PBS) was added to each well and the plate incubated at room temperature for 2 hours. The plate was washed three times with PBS/Tween 20 (0.05%) (Sigma). 100 μl of standard or sample diluted in blocking buffer/Tween 20 (0.05%) was added to each plate, which was then sealed with parafilm and incubated at 4°C overnight. The following day, the plate was washed four times with PBS/Tween. 100 ul of the detection antibody, Goat anti-human HGF antibody (Sigma), diluted to a concentration of 0.01 μg/ml in blocking buffer/Tween 20, was added to each well. After one hour incubation at room temperature, and 4 washes with PBS/Tween 20, 100 μl of avidin-horseradish peroxidase conjugated mouse anti-goat IgG (Jackson ImmunoReseach) diluted 1:1000 in blocking buffer/Tween 20 was added to each well. After 30 minutes incubation at room temperature and five washes with PBS/Tween 20, 200 μl of Sigma Fast OPD solution (Sigma) was added to each well. After 20 minutes, the plate was read at a wavelength of 405 nm using a microplate reader (Molecular Devices, Sunnyvale, CA). Data were analyzed using the Molecular Devices SOFTmax program.

### Cell migration

Cell migration assays were preformed according to product instructions (CSM Inc. Phoenix, AZ). Chilled cell manifolds were placed on Teflon-printed, precoated microscope slides, subdivided into 10 wells, and filled with ice-cold media. One μl of cell suspension (2500 cells) was added to each chamber and allowed to precipitate by gravity and adhere to the coverslip. After two hours, the manifold was moved to a cell incubator (37°C under 5% CO_2_) and allowed to reach growth temperature for 4 hours, at which point the cell sedimentation manifold was removed and the coverslip submerged in media, to which HGF was added. The area covered by cells was recorded at 2 hrs and subsequently every 24 hours. The 2 hr area was used as a reference point for all succeeding measurements. Results are presented as increases relative to this area.

### Protein sequencing of an HGF binding protein

The HGF and major co-immunoprecipitated product from C4-2 cell lysates were excised from the gel and transferred to a siliconized tube, washed and destained in 50% methanol overnight. The gel pieces were dehydrated in aceto-nitrile, rehydrated in 10 mM dithiothreitol (DTT) in 0.1 M ammonium bicarbonate, and reduced at room temperature for 30 minutes. The DTT solution was removed and the samples alkylated in 50 mM iodoacetamide, in 0.1 M ammonium bicarbonate, for 30 minutes at room temperature. Samples were then dehydrated again in aceto-nitrile, rehydrated in 0.1 M ammonium bicarbonate, dehydrated in aceto-nitrile and completely dried by vacuum centrifugation. Finally, samples were rehydrated for 10 minutes in 20 ng/ml trypsin in 50 mM ammonium bicarbonate on ice. Any excess trypsin solution was removed, and 50 mM ammonium bicarbonate added. Samples were digested overnight at 37°C and the sequences of generated peptides were identified by mass specectrometry.

### Cell lysis and Erk kinase assay

All cells were lysed in ice-cold lysis buffer (20 mMTris PH7.4, 40 mM NaCl, 20 mM beta-glycerophosphate, 2 mM EGTA, 1 mM sodium orthovanadate, 2 mM DTT, 2 mM PMSF, 1μg/ml aprotinin, and 1μg/ml aprotinin). The Map kinase assay was done with an assay kit (Upstate, Lake Placid, NY). The kinase reaction was started by addition of kinase reaction buffer that contains 2 mg/ml dephosphorylated myelin basic protein for each substrate, 20 mM MOPS (pH 7.2), 25 mM beta-Glycerophosphate, 5 mM EGTA, 0.4 mM MnCl_2_, 1 mM sodium orthovanadate, 1 mM dithiothreitol, 75 mM MgCl_2_, and 500 μM ATP. To prevent effects from other unknown kinases in the lysate, 20 μM PKC inhibitor peptide, 2 μM PKA inhibitor peptide, and 20 mM R24571 compound were added to the kinase reaction buffer. The reaction was incubated for 20 min at 30°C, terminated by the addition of the LDS sample buffer and loaded as aliquots for SDS-PAGE and immunoblot analyses. Membrane enriched fractions were purified as previously described [85].

### Immunoblot analyses

After centrifugation for 15 min at 15000 rpm in 4°C, the lysate supernatant was collected. Protein concentration was determined by BRC assay (BioRad, Hercules, CA). Immunoblotting was performed using the NOVEX (Invitrogen, Carlsbad, CA) system. Briefly, 7.5 μg of cell extracts and Erk kinase assay products were separated on 4–12% Tris glycine PAGE gels and transferred onto a PVDF membrane (Immoblin-P, Millipore, Billerica, MA). The membrane was blocked 1 h at room temperature with TBST blocking buffer [50 mM Tris-HCl (pH 8.0), 150 mM NaCl, 0.05% Tween 20, and 5% nonfat milk]. The membrane was incubated for 1 hr at room temperature with primary anti-phosph MBP polyclonal antibody, anti-Erk 1,2 antibody, anti-PKB antibody, and anti-phosph PKB antibody (Ser 473) in PBSN blocking buffer. A secondary antibody (horseradish peroxidase-anti-rabbit or mouse antibody) (Amersham Bioscience, Inc., Piscataway, NJ) at a 1:5000 dilution was used in PBSN blocking buffer and incubated for 1 hr at room temperature. ECL plus (Amersham Bioscience, Inc.) reagent was used for detection.

### Statistical analyses

Results were analyzed for statistical significance using the nonparametric Mann-Whitney U test, with significance at P < 0.05.

## Results

### HGF in stromal-conditioned media (SCM) regulates prostate cancer cell adhesive behaviors

**Figure 1:**
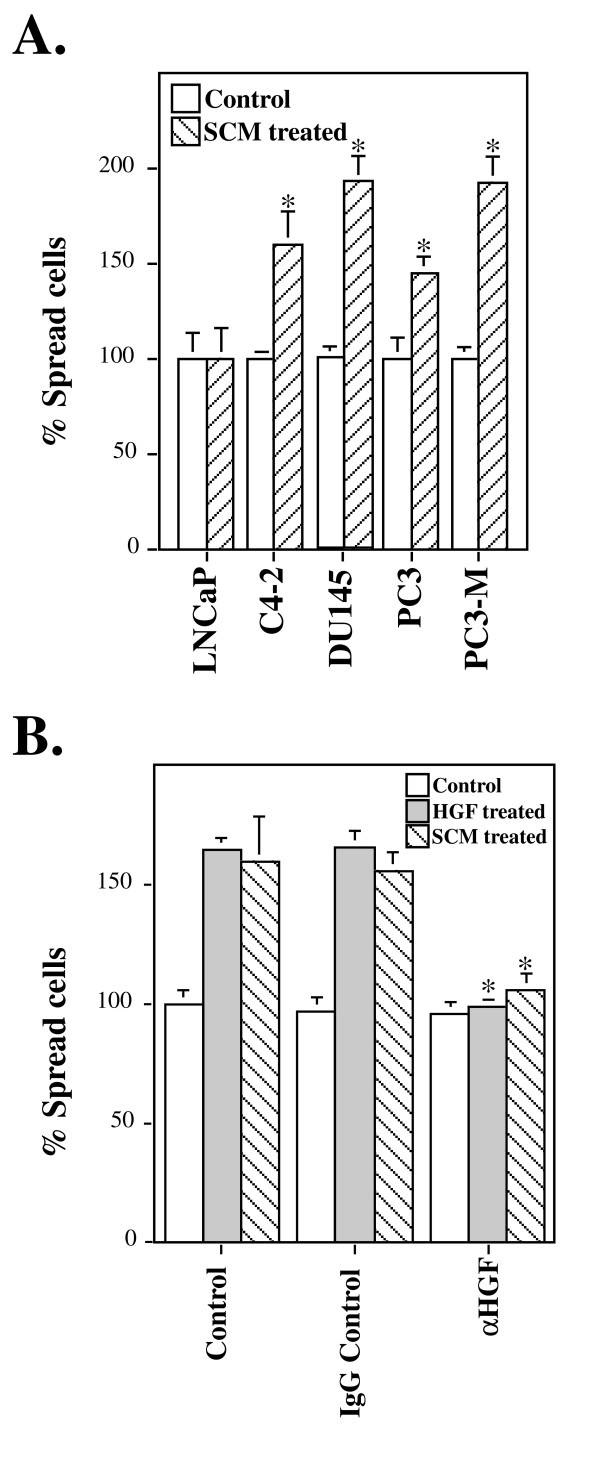
Prostate cancer cell lines compared for their cell spreading responses to treatment with stromal cell-conditioned media (SCM). (A) All cell lines, except LNCaP, increased cell spreading on laminin, following exposure to the SCM. (B) C4-2 cell attachment to laminin, following treatment with SCM or purified HGF. Cells were allowed to attach for 90 min., at which point ~25% of untreated C4-2 cells showed membrane protrusions. Induction of cell spreading, as seen for both SCM and purified HGF, is reversible by addition of anti-HGF antibody. Conditioned media from three primary stromal cell lines gave similar results (data not shown). Experimental cell spreading is shown as a percentage of control, untreated cells. Statistically significant differences from the control in each group were below P =< 0.05 (*). For each data point n = 6 or more.

Previously, our laboratory characterized cell responses to media conditioned by primary prostate stromal cells, and found differences between the responses of parental, non-metastatic, human prostate LNCaP cells and those of its lineage-derived, metastatic C4-2 subline [86]. Prostate SCM collected in serum-free conditions had little effect on LNCaP cells, but increased cell spreading of C4-2 cells on laminin-1 substrates by 150–200% (Figure 1A) [68]. C4-2 cells were not the only prostate cancer cells to respond this way to SCM; cell spreading also increased following SCM treatment of DU145 (brain metastatic human prostate cell line), PC3, and PC3M (a bone metastatic human prostate cell line of shared lineage). The SCM collected from five different primary cultures and three different hosts all had similar effects when incubated with C4-2 cells (data not shown). Many growth factors stimulate focal adhesion assembly and influence integrin activity [87], but we report here that these cell spreading effects of SCM can be ameliorated by anti-HGF antibodies (Figure 1B). Thus, the HGF in the SCM is a major regulator of cell adhesion and cell spreading.

**Figure 2:**
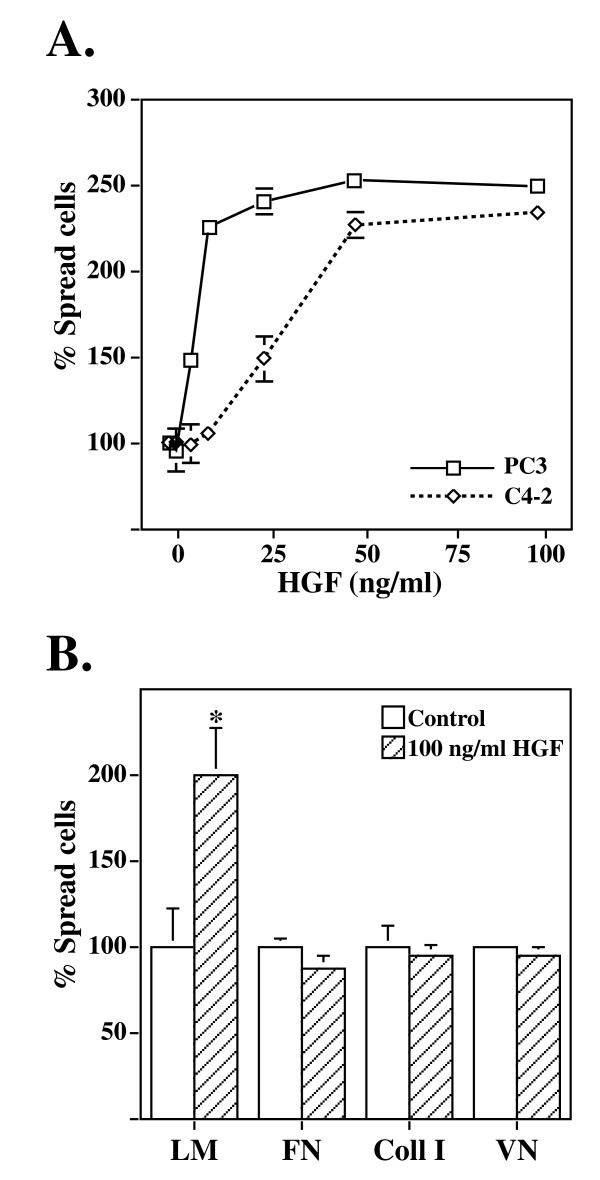
Effect of HGF on cell spreading and matrix requirements. (A) Cell spreading behaviors on laminin substrata for PC3 and C4-2 cells exposed to variable concentrations of purified HGF. Because the cells differ in untreated attachment speeds, experiments were terminated at 30 minutes for PC3 cells and 90 minutes for C4-2 cells. At these times, with high concentrations of HGF, filopodia and lamelipodia were visible in approximately 90% of PC3 cells and 65% of C4-2 cells. Both cell lines show a concentration-dependent increase in cell spreading. (B) HGF induction of C4-2 cell spreading is matrix-dependent, with spreading increasing only on laminin-1 matrix (laminin, Fibronectin, Collagen I, and Vitronectin are LM, FN, CollI and VN, respectively). Experimental cell spreading is shown as a percentage of control, untreated cells. Statistically significant differences from the control in each group were below P =< 0.05 (*). For each data point n = 6 or more.

**Figure 3:**
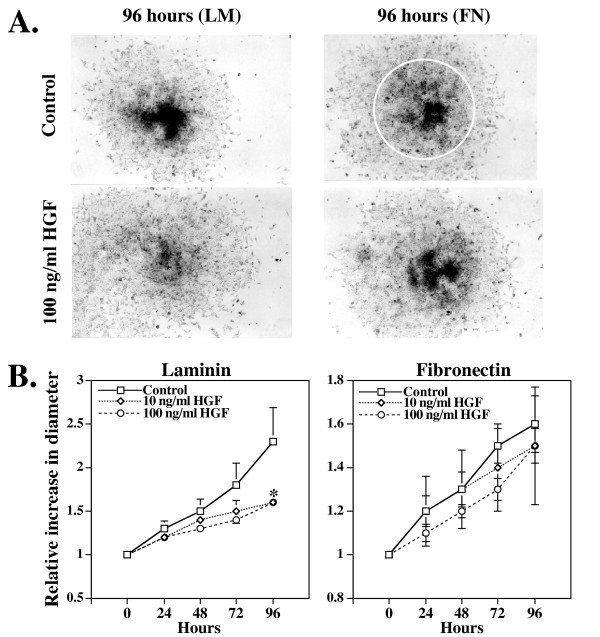
Time course and quantification of C4-2 migratory behaviors. C4-2 cells were plated in a defined circular area on laminin or fibronectin substrata, with different concentrations of HGF in the media. (A) Migration at 96 hr. time point, with 0 or 100 ng/ml of HGF in the medium. (B) Time-dependent migration, in the presence of HGF, on different matrices. All values are normalized to the cell area at 0 hr. time point.

To measure HGF amounts present in the SCM, we used ELISA's and found HGF to range from 14 to 24 ng/ml (Table 1). Cell spreading responses to HGF are concentration-dependent for both PC3 and C4-2 prostate cancer cell lines, but an effective dose 50 (EC50) for PC3 cells is achieved at a lower concentration (5 ng/ml) than for C4-2 cells (30 ng/ml) (Figure 2A). These differences in maximal stimulation could be due to synergism between HGF and other growth factors in the SCM, to the combined presence of both met and nucleolin in PC3 cells, and/or to differences in nucleolin and met affinities for HGF. HGF-enhanced C4-2 cell spreading was substrate dependent and observed only on laminin-1 (LM) substrate (Figure 2B), not on fibronectin (FN), collagen-1 (Col-1) or vitronectin (VN). C4-2 cell spreading and cell migration respond inversely to HGF treatment; that is, cell spreading is enhanced, whereas cell locomotion is decreased on laminin. The inhibitory effects of HGF on cell migration were observed at 10 ng/ml (Figure 3A and 3B). Like the cell spreading effects, migration inhibition was seen only on a laminin-1 substrate, not on FN. HGF migration responses could not be explained by cell proliferation, because C4-2 cell cycle progression appeared unaffected by HGF (data not shown). Furthermore, all cellular adhesion could be block by either α_6 _or β_1 _integrin function blocking antibodies and no changes in surface expression of laminin binding integrins were observed (data not shown).

### HGF-induced cell adhesion and migration responses are mediated by Met-independent receptors

**Figure 4:**
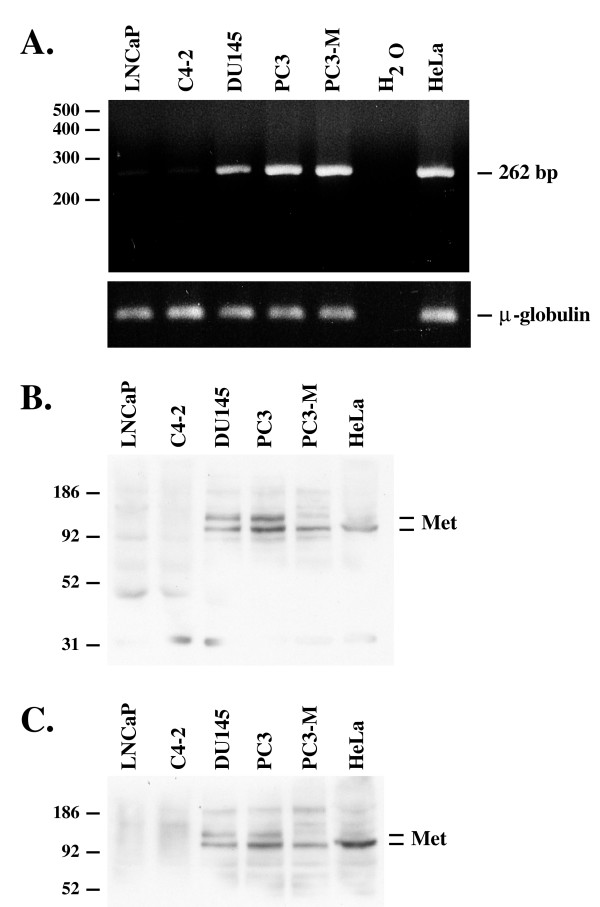
Expression of met in prostate cancer cell lines. (A) Met RT-PCR detection of six transcripts in prostate cell lines. Amplified fragments of control μ-globulin and the cytoplasmic domain of human met are shown on an ethidium-bromide stained agarose gel. The length of the h-met PCR product was 262 bp. Product levels were close to undetectable in LNCaP and C4-2 samples. (B) Western blot analysis of total cell lysates from same prostate cell lines and HeLa cells. Equal amounts of cell lysate were separated and immunoblotted with antibodies against either the extracellular (B) or cytoplasmic (C) domains of Met. Met was detected by characteristic double bands in all cell lines except LNCaP and C4-2.

Using RT-PCR, we assess met expression levels in prostate cancer cell lines of different metastatic potential (LNCaP, C4-2, PC3 and DU145). Met receptor transcript (262 bp) was present in DU145, PC3, PC3M and HeLa cells, but absent or at very low levels in both LNCaP and C4-2 cells (Figure 4A). These results were further confirmed by Western blot analyses. Doublets of c-Met were observed, with similar results for both the N-terminal and C-terminal Met antibodies, used to check for possible Met isoforms differing in their cytoplasmic tails (Figure 4B and 4C).

**Figure 5:**
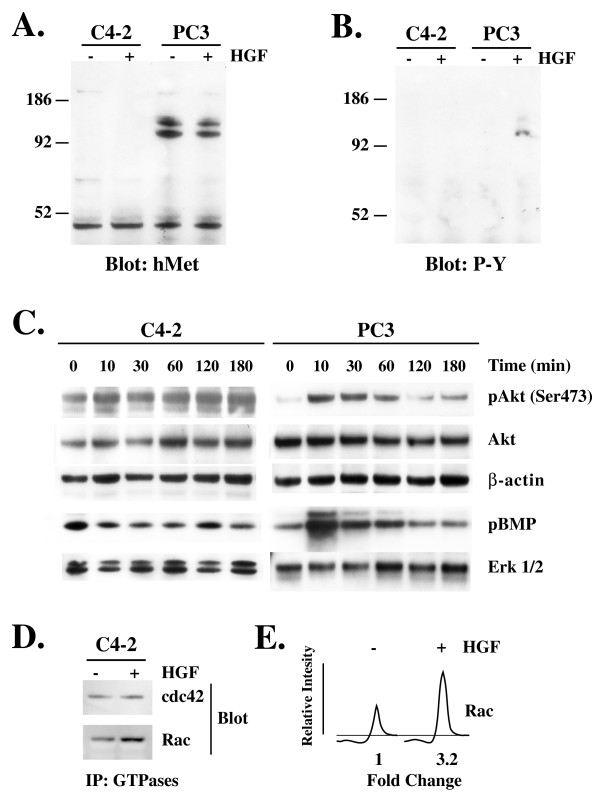
(A, B) Immunoprecipitation of Met in C4-2 and PC3 with and without activation by HGF. (C) Time-dependent activation of both Erk and Akt is visible in PC3 cells but not C4-2. (D, E) Rac is activated but not cdc42 upon HGF stimulation in C4-2 cells, as seen by immunoprecipitation of active GTPases.

The HGF-Met signaling cascade is well described and known to regulate invasion and metastasis, as well as cell proliferation, survival, differentiation and branching morphogenesis. Interactions between active HGF and Met result in αβ heterodimer formation, trans-autophosphorylation, and the recruitment of signaling intermediates [21,33]. We searched for Akt and Erk phosphorylation in both PC3 and C4-2 cells, and confirmed that C4-2 cells do not respond to HGF with a functional Met signaling system. Following HGF stimulation, Met is phosophorylated and activated only in PC3 cells, not C4-2 (Figure 5A and 5B). Furthermore, both Akt and Erk are activated in a time-dependent manner in PC3 cells, but not in C4-2 cells (Figure 5C). These findings disagree with a previous report of a weak response to HGF, which may have been due to activation of the PI3-kinase/Akt pathway by cell-substrate adhesion that was further enhanced by HGF/SF stimulation [88]. Our static stimulation of serum-starved cells reveals clear enhancement of both Akt and Erk in PC3, but not C4-2 cells. Since both RT-PCR and Western blot were negative for met/Met in the LNCaP progression model, their dose-dependent responses to HGF stimulation suggest a Met-independent mechanism. This is further shown by the activation of Rac upon HGF stimulation in the C4-2 cells (Figure 5D and 5E). Together, these results correlate well with our cell-spreading and increased membrane ruffling data and further argue for a Met-independent pathway for HGF response in C4-2 cells.

**Figure 6:**
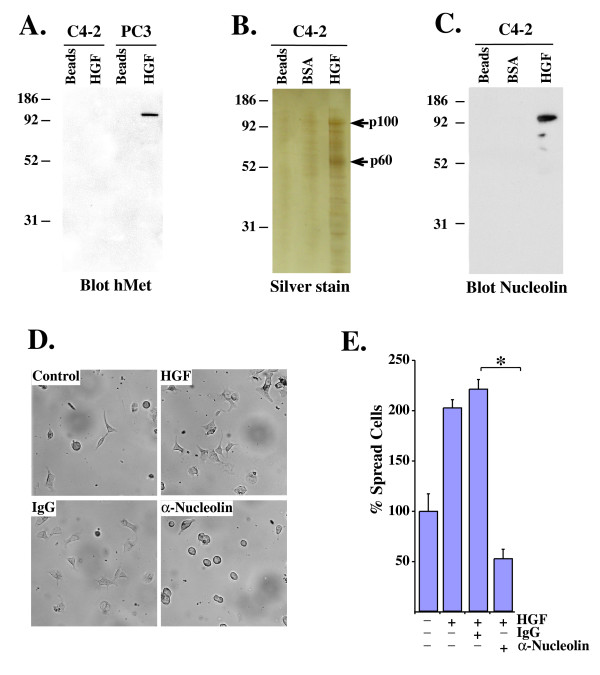
Precipitation of HGF binding proteins from C4-2 and PC3 cells, using beads coated with HGF or BSA. (A) Met was precipitated in the PC3 cell line, while (B) in the C4-2 cell line two bands were precipitated using HGF coated beads but not with BSA control beads. The lower major band is HGF itself; the upper 100 kDa band was purified and sequenced. Western-blot analyses (C) and sequencing of the upper band confirmed it to be nucleolin (see table 2). (D) The importance of nucleolin to HGF-stimulated spreading on laminin substrata was apparent when antibodies to nucleolin reduced the effect of HGF (E). The cell spreading behaviors were quantified and presented as the mean of triplicate experiments. Statistically significant differences from the control were at P =< 0.005 (*).

Immunoprecipitation of PC3 and C4-2 total cell lysates further confirms that Met is present in HGF-stimulated PC3 cells and absent in C4-2 cells (Figure. 6A). HGF was immobilized on agarose beads and incubated with cell lysates. Immunoprecipitated product was then separated and stained with an antibody against the Met cytoplasmic domain. Control beads pulled down no detectable Met protein from either PC3 or C4-2 lysates, as assayed by both silver staining and immunoblotting (Figure 6A and 6B). Note that the silver-stained, immunoprecipitated products of C4-2 cells contained two major bands of approximately 60 kDa and 100 kDa. The lower band represents HGF itself, as identified by Western blot (data not shown), whereas the higher band was micro-sequenced and identified as nucleolin, with an expected size of 98 kDa (Table 2).

### Nucleolin is an HGF binding partner in the C4-2 prostate cancer cell line

**Figure 7:**
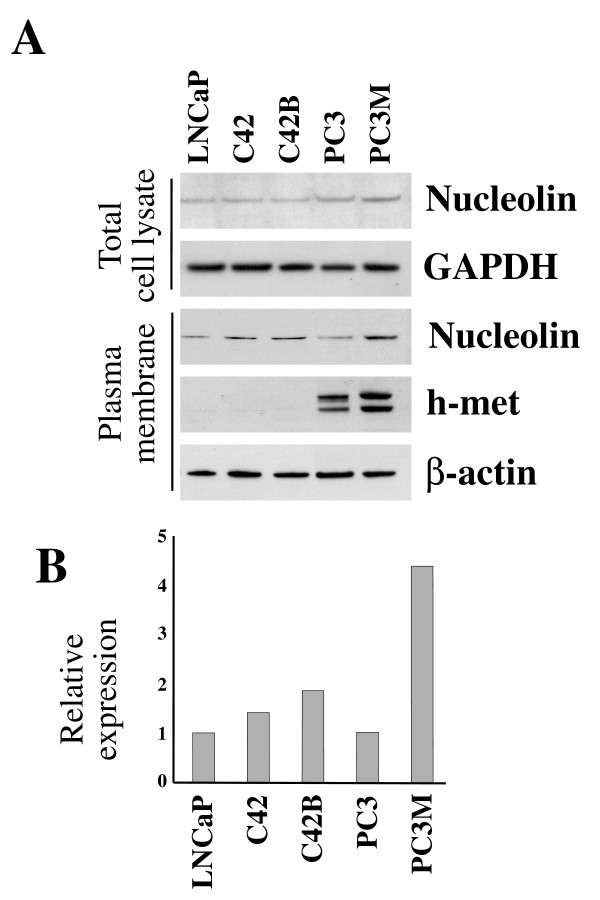
Nucleolin and Met expression profiles during disease progression, as modeled across LNCaP and PC3 prostate cancer cell lines. (A) Comparison of protein levels in total cell lysate and plasma membrane fractions by Western blotting. (B) Normalizing membrane nucleolin levels to levels in the less-advanced parental cell lines of each progression model, reveals that membrane nucleolin levels are higher than parental in both C4-2 and PC3M. Nucleolin levels were 30% higher in prostate parental cells compared to normal (p69 and RWPE-2) prostate epithelial cells (data not shown).

Of the 98 kDa band isolated from total C4-2 cell lysates (by columns of HGF-coated agarose beads), a total of 24 peptides were sequenced, corresponding to approximately 30% of the protein sequence of nucleolin, with 100% identity match to the previously published protein sequence (NCBI#4885511; Table 2). The identity of the 100 kDa protein was further confirmed by Western blotting to nucleolin (Figure. 6C). No other sequence homologies were found, suggesting that nucleolin is the major protein in the 100 kDa band, and that nucleolin is the major binding partner for HGF in C4-2 cells. Most compellingly, antibodies against nucleolin were able to block the cell spreading phenotype observed in HGF-treated cells in our cell attachment assay system (Figure 6D and 6E); in other words, Met-negative C4-2 cells increased cell spreading behavior when treated with HGF, and this response could be abolished by the addition of antibodies against the nucleolin protein (or HGF or specific integrin subunits, data not shown). Although we do not see any changes in the total nucleolin expression levels in the cells, we do see an increase in the membrane-associated nucleolin in both the LNCaP and PC3 progression models (Figure 7). This strengthens the link between nucleolin and HGF function, at the same time that it argues for cell surface localization of the nucleolin protein during cancer progression.

## Discussion

We have used prostate cancer cell lines and stromal-conditioned media to study the regulatory interplay between prostate epithelial and stromal cells during prostate cancer progression, and have now begun to tease apart the relationships between stromally-derived HGF signal, Met signal reception, integrin-based cell adhesive responses, and a new HGF binding partner, the nucleolin protein. We report here: 1) that it is the HGF in stromal-conditioned media that affects C4-2 metastatic prostate cancer cell adhesive behavior, 2) that this action is through the integrins, and 3) that the dose-dependent response of Met-negative C4-2 cells to HGF treatment can be abolished through the addition of antibodies against the nucleolin protein.

### HGF response without Met and with nucleolin

A Met-independent pathway acting in the absence of Met and/or alongside Met could increase the variety of possible cell responses to HGF. Classically, the binding of HGF to Met induces receptor dimerization and phosphorylation of two conserved tyrosine sites; when these sites are mutated, mice show similar phenotypes to those with HGF or Met knockouts [89]. But C4-2 cells, which lack this signaling pair, could be revealing spreading behaviors due to low-affinity HGF sites that allow response to higher levels of HGF (compared to Met-expressing PC3 cells; Figure 2). Past research on HGF affinity has identified some binding sites in hepatocytes with 10-fold lower affinity [90]. HGF interactions with proteoglycans and CD44 is linked to enhanced Met signaling and possible Met autophosphorylation [91,92]. Pollack et al. [93] suggest that in Madin Darby canine kidney (MDCK) cells, low-affinity binding of HGF in the presence of the Met receptor may alter downstream responses. Thus, we are certainly not the first to suggest either additional HGF receptors, or HGF binding to heparin and oligosaccharide signaling systems [94,95]. Our study is unique, however, in our identification of the HGF binding partner nucleolin.

Nucleolin, originally called C23 [96], was first characterized as an abundant nuclear protein with a role in ribosome biogenesis [69,70]. Because nucleolin synthesis correlates positively with cell growth, the especially high expression of nucleolin in tumor cells is not surprising [97]. Nucleolin is further a major regulatory and phosphorylation target following androgen treatment [98,99]. Nucleolin's *in vivo *localization to the cell surface in aggressive tumor cells [77,78,99,100] agrees with our *in vitro *finding of membrane localization in both LNCaP and PC3 progression model cell lines (Figure 7). This membrane localization has recently generated interest in the use of nucleolin as a tumor marker and therapeutic target. A nucleolin functional inhibitor called AGRO100 (a guanine-rich oligonucletide) has been successful in Phase I clinical trials, and Phase II trials are underway [77,78,101]. Further evidence of nucleolin's role in cancer progression comes from our unrelated, *in vivo *bio-panning study. While bio-panning with a 12-mer peptide phage-display library, a peptide (designated L13) was found to bind with high specificity to murine bone marrow. When human bone marrow endothelial cell lysates were passed across an L13 column, the major species eluted was found to be nucleolin ([102]*and unpublished observations*). Indeed, a tumor homing peptide, F3, that binds specifically to tumor endothelial cells was described by Christian et al. [75] as interacting directly with nucleolin. Together, there is now clear evidence of nucleolin's involvement in cancer cell behavior and response to HGF, although this protein's direct and/or indirect functions remain to be discovered.

### Nucleolin on the cell surface

Nucleolin is named for the fact that it makes up as much as 10% of the total nucleolar protein [103], but it also functions as a shuttle protein between the cytoplasm and the nucleus, is found in clusters associated with the actin cytoskeleton [104-106], and is sensitive to cytochalasin D [104]. Nucleolin is present on the cell surfaces of a variety of cell types [71,107,108], and surface-expressed nucleolin appears to interact with an array of other proteins, including viral proteins during infections [109,110], βFGF and Midkine [111], and laminin-1 [112,113].

A three-way interaction between nucleolin, HGF, and integrins is suggested by our ability, in short-term assays, to inhibit HGF's cell spreading effects using anti-nucleolin antibodies (Figure 6D); Nucleolin inhibition, alone, however, in the absence of HGF stimulation, does not alter C4-2 laminin adhesion (data not shown). Also, nucleolin by itself is not able to sustain laminin adhesion, as we were able to completely block cell spreading under stimulated and un-stimulated conditions, using function-blocking antibodies to either β_1 _or α_6 _integrin (data not shown). Yu et al. [114] studied the cellular distribution of nucleolin after stimulation with ECM proteins, and report that, following laminin stimulation, nucleolin translocates from the cytoplasm to the nucleus and stimulates cell proliferation. Following HGF stimulation, we do not detect changes in laminin-binding integrin expression profiles (data not shown), but we have previously seen changes in integrin function (not expression) in these same prostate cells [68]. Also possible is the movement of additional nucleolin protein to the cell surface, providing direct increase in laminin adhesion [72,112,114]. Nucleolin may function as a generic GF-HS binding receptor, linking HGF and integrin function. Like syndecan, nucleolin has been found to bind HGF's heparin-binding domain, thereby sequestering HGF on the cell surface. In our studies, nucleolin does not co-immunoprecipitate with other heparin-binding growth factors (data not shown), but HGF-Nucleolin interactions do respond to heparin competition (data not shown). We are currently searching for other components in nucleolin-HGF interactions using BIAcore. The findings we report here, together with the nucleolin-focused independent clinical trial underway [77,78], introduce nucleolin as an attractive target for therapeutic regulation of prostate cancer, although the details of its position in cancer cell HGF signaling remain to be found.

### Additional components in HGF/nucleolin interactions

We sought to begin placing nucleolin in or beside well-described HGF-met signaling pathways affecting integrin-based cell behaviors. Cell spreading and membrane ruffling behaviors seen in C4-2's met-independent HGF cell responses led us to look first at small GTPases (Figure 5). We found that Rac is activated by HGF treatment of met-negative C4-2 cells (Figure 5D,E). Rho, Rac and cdc42 are part of the Ras small GTPase superfamily, and play key roles in regulating cell shape, contraction, adhesion, motility, and proliferation [115-117]. Members of this GTPase superfamily are also known molecular triggers to response cascades following growth factor stimulation ([118] and references within). Rac, in particular is a regulator of the superoxide generating NADPH oxidase in phagocytes [119]. The production of reactive oxygen species (ROS) mediates activation of NF-κB-dependent gene expression, essential for tumorigenesis and metastasis.

## Conclusion

We have found evidence that HGF secreted by prostate stromal cells regulates prostate cancer cell adhesive behaviors, even in cells that lack Met, the one known HGF receptor. Further, we report that the nucleolin protein expressed on the surfaces of these prostate cancer cells (in increasing levels during disease progression) is involved in the HGF regulatory interplay between stromal and epithelial cells within the prostate, and possibly also at sites of metastasis. A family of drugs targeting the nucleolin protein are being developed for cancer therapy; these AGRO100 drugs (named for Guanine-Rich Oligonucleotides) reportedly acted against cultured cell lines from several different cancers and successfully stabilized disease in six of nine patients in phase I clinical trials. They are now in phase II trials [77,78]. We have independently identified nucleolin protein as having a role in cancer progression, and have now linked nucleolin's function to prostate cancer cell reception of Hepatocyte Growth Factor (HGF) and integrin-based cell adhesive behaviors.

## Abbreviations

Abbreviations are defined in the text

## Competing interests

The author(s) declare that they have no competing interests.

## Authors' contributions

SI carried out the signaling pathway analyses upon HGF stimulation in figure 5C. RAS: carried out PCR analyses of met expression, and provided comments on the manuscript draft. RD and LWKC provided concept ideas in the early part of this work. They also provided comments on the manuscript. AT, MB and ME have performed the substantial body of work presented in this manuscript. Each contributed to the writing of the manuscript. All authors read and approved the final manuscript.

## Pre-publication history

The pre-publication history for this paper can be accessed here:



## Figures and Tables

**Table 1:** ELISA measurements of HGF concentrations in stromal-conditioned media from normal prostate stromal cells after 24 hr. culture

Stroma 1	18.5 (4.6)
Stroma 2	24.1 (2.3)
Stroma 3	16.4 (2.0)
Stroma 4	13.9 (1.5)
Stroma 5	22.6 (3.2)

**Table 2:** 24 peptides were sequenced from cell lysates, based on co-immunoprecipitation with purified HGF protein. All peptides showed 100% sequence homology to previously cloned nucleolin (NCBI#4885511). Numbers within the Table refer to the peptide's location within the published nucleolin sequence.

72–79	VAVATPAK
72–80	VAVATPAKK
296–318	QKVEGTEPTTAFNLFVGNLNFNK
325–333	TGISDVFAK
334–342	NDLAVVDVR
348–362	KEGYVDFESAEDLEK
349–362	FGYVDFESAEDLEK
363–370	ALELTGLK
404–410	VTQDELK
404–420	VTQDELKEVFEDAAEIR
411–420	EVFEDAAEIR
428–437	SKGIAYIEFK
430–437	GIAYIEFK
438–449	TEADAEKTFEEK
450–457	QGTEIDGR
458–467	SISLYYTGEK
478–486	NSTWSGESK
522–537	SKGYAFIEFASFEDAK
525–537	GYAFIEFASFEDAK
555–561	LELQGPR
578–597	GLSEDTTEETLKESFDGSVR
611–624	GFGFVDFNSEEDAK
625–636	EAMEDGEIDGNK
625–645	EAMEDGEIDGNKVTLDWAKPK

## References

[B1] Chung LW, Hsieh CL, Law A, Sung SY, Gardner TA, Egawa M, Matsubara S, Zhau HE (2003). New targets for therapy in prostate cancer: modulation of stromal-epithelial interactions. Urology.

[B2] Tuxhorn JA, Ayala GE, Rowley DR (2001). Reactive stroma in prostate cancer progression. J Urol.

[B3] McCawley LJ, Matrisian LM (2001). Tumor progression: defining the soil round the tumor seed. Curr Biol.

[B4] Liotta LA, Kohn EC (2001). The microenvironment of the tumour-host interface. Nature.

[B5] David Roodman G (2003). Role of stromal-derived cytokines and growth factors in bone metastasis. Cancer.

[B6] Chung LW, Baseman A, Assikis V, Zhau HE (2005). Molecular insights into prostate cancer progression: the missing link of tumor microenvironment. J Urol.

[B7] Nemeth JA, Lee C (1996). Prostatic ductal system in rats: regional variation in stromal organization. Prostate.

[B8] Cunha GR, Battle E, Young P, Brody J, Donjacour A, Hayashi N, Kinbara H (1992). Role of epithelial-mesenchymal interactions in the differentiation and spatial organization of visceral smooth muscle. Epithelial Cell Biol.

[B9] Edlund M, Sung SY, Chung LW (2004). Modulation of prostate cancer growth in bone microenvironments. J Cell Biochem.

[B10] Sung SY, Chung LW (2002). Prostate tumor-stroma interaction: molecular mechanisms and opportunities for therapeutic targeting. Differentiation.

[B11] Liotta LA, Rao CN (1986). Tumor invasion and metastasis. Monogr Pathol.

[B12] Ma H, Calderon TM, Kessel T, Ashton AW, Berman JW (2003). Mechanisms of hepatocyte growth factor-mediated vascular smooth muscle cell migration. Circ Res.

[B13] Santos OF, Moura LA, Rosen EM, Nigam SK (1993). Modulation of HGF-induced tubulogenesis and branching by multiple phosphorylation mechanisms. Dev Biol (N Y 1985).

[B14] Birchmeier C, Birchmeier W, Gherardi E, Vande Woude GF (2003). Met, metastasis, motility and more. Nat Rev Mol Cell Biol.

[B15] Andermarcher E, Surani MA, Gherardi E (1996). Co-expression of the HGF/SF and c-met genes during early mouse embryogenesis precedes reciprocal expression in adjacent tissues during organogenesis. Dev Genet.

[B16] Weidner KM, Sachs M, Birchmeier W (1993). The Met receptor tyrosine kinase transduces motility, proliferation, and morphogenic signals of scatter factor/hepatocyte growth factor in epithelial cells. J Cell Biol.

[B17] Park M, Dean M, Kaul K, Braun MJ, Gonda MA, Vande Woude G (1987). Sequence of MET protooncogene cDNA has features characteristic of the tyrosine kinase family of growth-factor receptors. Proc Natl Acad Sci U S A.

[B18] Cooper CS, Park M, Blair DG, Tainsky MA, Huebner K, Croce CM, Vande Woude GF (1984). Molecular cloning of a new transforming gene from a chemically transformed human cell line. Nature.

[B19] Bottaro DP, Rubin JS, Faletto DL, Chan AM, Kmiecik TE, Vande Woude GF, Aaronson SA (1991). Identification of the hepatocyte growth factor receptor as the c-met proto-oncogene product. Science.

[B20] Naldini L, Weidner KM, Vigna E, Gaudino G, Bardelli A, Ponzetto C, Narsimhan RP, Hartmann G, Zarnegar R, Michalopoulos GK (1991). Scatter factor and hepatocyte growth factor are indistinguishable ligands for the MET receptor. EMBO Journal.

[B21] Knudsen BS, Edlund M (2004). Prostate cancer and the met hepatocyte growth factor receptor. Adv Cancer Res.

[B22] Lyon M, Deakin JA, Mizuno K, Nakamura T, Gallagher JT (1994). Interaction of hepatocyte growth factor with heparan sulfate. Elucidation of the major heparan sulfate structural determinants. J Biol Chem.

[B23] Hartmann G, Prospero T, Brinkmann V, Ozcelik C, Winter G, Hepple J, Batley S, Bladt F, Sachs M, Birchmeier C (1998). Engineered mutants of HGF/SF with reduced binding to heparan sulphate proteoglycans, decreased clearance and enhanced activity in vivo. Curr Biol.

[B24] Lamszus K, Joseph A, Jin L, Yao Y, Chowdhury S, Fuchs A, Polverini PJ, Goldberg ID, Rosen EM (1996). Scatter factor binds to thrombospondin and other extracellular matrix components. Am J Pathol.

[B25] Weidner KM, Behrens J, Vandekerckhove J, Birchmeier W (1990). Scatter factor: molecular characteristics and effect on the invasiveness of epithelial cells. J Cell Biol.

[B26] Rosen EM, Goldberg ID, Kacinski BM, Buckholz T, Vinter DW (1989). Smooth muscle releases an epithelial cell scatter factor which binds to heparin. In Vitro Cell Dev Biol.

[B27] Gmyrek GA, Walburg M, Webb CP, Yu HM, You X, Vaughan ED, Vande Woude GF, Knudsen BS (2001). Normal and malignant prostate epithelial cells differ in their response to hepatocyte growth factor/scatter factor. Am J Pathol.

[B28] Humphrey PA, Zhu X, Zarnegar R, Swanson PE, Ratliff TL, Vollmer RT, Day ML (1995). Hepatocyte growth factor and its receptor (c-MET) in prostatic carcinoma. Am J Pathol.

[B29] Pisters LL, Troncoso P, Zhau HE, Li W, von Eschenbach AC, Chung LW (1995). c-met proto-oncogene expression in benign and malignant human prostate tissues. J Urol.

[B30] Xue Y, Smedts F, Ruijter ET, Debruyne FM, de la Rosette JJ, Schalken JA (2001). Branching activity in the human prostate: a closer look at the structure of small glandular buds. Eur Urol.

[B31] Xue Y, Sonke G, Schoots C, Schalken J, Verhofstad A, de la Rosette J, Smedts F (2001). Proliferative activity and branching morphogenesis in the human prostate: a closer look at pre- and postnatal prostate growth. Prostate.

[B32] Rosen EM, Nigam SK, Goldberg ID (1994). Scatter factor and the c-met receptor: a paradigm for mesenchymal/epithelial interaction. J Cell Biol.

[B33] Rosario M, Birchmeier W (2003). How to make tubes: signaling by the Met receptor tyrosine kinase. Trends Cell Biol.

[B34] Birchmeier C, Gherardi E (1998). Developmental roles of HGF/SF and its receptor, the c-Met tyrosine kinase. Trends Cell Biol.

[B35] Brinkmann V, Foroutan H, Sachs M, Weidner KM, Birchmeier W (1995). Hepatocyte growth factor/scatter factor induces a variety of tissue-specific morphogenic programs in epithelial cells. J Cell Biol.

[B36] McNeal JE (1965). Morphogenesis of prostatic carcinoma. Cancer.

[B37] Bonkhoff H (2001). Morphogenesis of prostate cancer. Eur Urol.

[B38] Lee JH, Han SU, Cho H, Jennings B, Gerrard B, Dean M, Schmidt L, Zbar B, Vande Woude GF (2000). A novel germ line juxtamembrane Met mutation in human gastric cancer. Oncogene.

[B39] Di Renzo MF, Olivero M, Martone T, Maffe A, Maggiora P, Stefani AD, Valente G, Giordano S, Cortesina G, Comoglio PM (2000). Somatic mutations of the MET oncogene are selected during metastatic spread of human HNSC carcinomas. Oncogene.

[B40] Schmidt L, Junker K, Nakaigawa N, Kinjerski T, Weirich G, Miller M, Lubensky I, Neumann HP, Brauch H, Decker J (1999). Novel mutations of the MET proto-oncogene in papillary renal carcinomas. Oncogene.

[B41] Jeffers M, Schmidt L, Nakaigawa N, Webb CP, Weirich G, Kishida T, Zbar B, Vande Woude GF (1997). Activating mutations for the met tyrosine kinase receptor in human cancer. Proc Natl Acad Sci U S A.

[B42] Lengyel E, Prechtel D, Resau JH, Gauger K, Welk A, Lindemann K, Salanti G, Richter T, Knudsen B, Vande Woude GF (2005). C-Met overexpression in node-positive breast cancer identifies patients with poor clinical outcome independent of Her2/neu. Int J Cancer.

[B43] Knudsen BS, Gmyrek GA, Inra J, Scherr DS, Vaughan ED, Nanus DM, Kattan MW, Gerald WL, Vande Woude GF (2002). High expression of the Met receptor in prostate cancer metastasis to bone. Urology.

[B44] Jeffers M, Fiscella M, Webb CP, Anver M, Koochekpour S, Vande Woude GF (1998). The mutationally activated Met receptor mediates motility and metastasis. Proc Natl Acad Sci U S A.

[B45] Jeffers M, Rong S, Woude GF (1996). Hepatocyte growth factor/scatter factor-Met signaling in tumorigenicity and invasion/metastasis. J Mol Med.

[B46] Watanabe M, Fukutome K, Kato H, Murata M, Kawamura J, Shiraishi T, Yatani R (1999). Progression-linked overexpression of c-Met in prostatic intraepithelial neoplasia and latent as well as clinical prostate cancers. Cancer Lett.

[B47] Kurimoto S, Moriyama N, Horie S, Sakai M, Kameyama S, Akimoto Y, Hirano H, Kawabe K (1998). Co-expression of hepatocyte growth factor and its receptor in human prostate cancer. Histochem J.

[B48] Tsuka H, Mori H, Li B, Kanamaru H, Matsukawa S, Okada K (1998). Enhanced hepatocyte growth factor level in human prostate cancer treated with endocrine therapy. Int J Oncol.

[B49] Tsuka H, Mori H, Li B, Kanamaru H, Matsukawa S, Okada K (1998). Expression of c-MET/HGF receptor mRNA and protein in human non-malignant and malignant prostate tissues. Int J Oncol.

[B50] Nagakawa O, Murakami K, Yamaura T, Fujiuchi Y, Murata J, Fuse H, Saiki I (2000). Expression of membrane-type 1 matrix metalloproteinase (MT1-MMP) on prostate cancer cell lines. Cancer Lett.

[B51] Qadan LR, Perez-Stable CM, Schwall RH, Burnstein KL, Ostenson RC, Howard GA, Roos BA (2000). Hepatocyte growth factor and vitamin D cooperatively inhibit androgen-unresponsive prostate cancer cell lines. Endocrinology.

[B52] Nishimura T, Toda S, Mitsumoto T, Oono S, Sugihara H (1998). Effects of hepatocyte growth factor, transforming growth factor-beta1 and epidermal growth factor on bovine corneal epithelial cells under epithelial-keratocyte interaction in reconstruction culture. Exp Eye Res.

[B53] Nishimura K, Kitamura M, Miura H, Nonomura N, Takada S, Takahara S, Matsumoto K, Nakamura T, Matsumiya K (1999). Prostate stromal cell-derived hepatocyte growth factor induces invasion of prostate cancer cell line DU145 through tumor-stromal interaction. Prostate.

[B54] Nishimura K, Matsumiya K, Miura H, Tsujimura A, Nonomura N, Matsumoto K, Nakamura T, Okuyama A (2003). Effects of hepatocyte growth factor on urokinase-type plasminogen activator (uPA) and uPA receptor in DU145 prostate cancer cells. Int J Androl.

[B55] Nishimura K, Kitamura M, Takada S, Nonomura N, Tsujimura A, Matsumiya K, Miki T, Matsumoto K, Okuyama A (1998). Regulation of invasive potential of human prostate cancer cell lines by hepatocyte growth factor. Int J Urol.

[B56] Chan AM, Rubin JS, Bottaro DP, Hirschfield DW, Chedid M, Aaronson SA (1991). Identification of a competitive HGF antagonist encoded by an alternative transcript. Science.

[B57] Chan A, Rubin J, Bottaro D, Hirschfield D, Chedid M, Aaronson SA (1993). Isoforms of human HGF and their biological activities. EXS.

[B58] Ferracini R, Longati P, Naldini L, Vigna E, Comoglio PM (1991). Identification of the major autophosphorylation site of the Met/hepatocyte growth factor receptor tyrosine kinase. J Biol Chem.

[B59] Gandino L, Longati P, Medico E, Prat M, Comoglio PM (1994). Phosphorylation of serine 985 negatively regulates the hepatocyte growth factor receptor kinase. J Biol Chem.

[B60] Prat M, Crepaldi T, Gandino L, Giordano S, Longati P, Comoglio P (1991). C-terminal truncated forms of Met, the hepatocyte growth factor receptor. Mol Cell Biol.

[B61] Rodrigues GA, Naujokas MA, Park M (1991). Alternative splicing generates isoforms of the met receptor tyrosine kinase which undergo differential processing. Mol Cell Biol.

[B62] Rodrigues GA, Park M (1993). Isoforms of the met receptor tyrosine kinase. EXS.

[B63] Wordinger RJ, Clark AF, Agarwal R, Lambert W, Wilson SE (1999). Expression of alternatively spliced growth factor receptor isoforms in the human trabecular meshwork. Invest Ophthalmol Vis Sci.

[B64] Meiners S, Brinkmann V, Naundorf H, Birchmeier W (1998). Role of morphogenetic factors in metastasis of mammary carcinoma cells. Oncogene.

[B65] Birchmeier W, Brinkmann V, Niemann C, Meiners S, DiCesare S, Naundorf H, Sachs M (1997). Role of HGF/SF and c-Met in morphogenesis and metastasis of epithelial cells. Ciba Found Symp.

[B66] Kim SJ, Shiba E, Tsukamoto F, Izukura M, Taguchi T, Yoneda K, Tanji Y, Kimoto Y, Takai SI (1998). The expression of urokinase type plasminogen activator is a novel prognostic factor in dukes B and C colorectal cancer. Oncol Rep.

[B67] Nakamura T, Nishizawa T, Hagiya M, Seki T, Shimonishi M, Sugimura A, Tashiro K, Shimizu S (1989). Molecular cloning and expression of human hepatocyte growth factor. Nature.

[B68] Edlund M, Miyamoto T, Sikes RA, Ogle R, Laurie GW, Farach-Carson MC, Otey CA, Zhau HE, Chung LW (2001). Integrin expression and usage by prostate cancer cell lines on laminin substrata. Cell Growth Differ.

[B69] Ginisty H, Sicard H, Roger B, Bouvet P (1999). Structure and functions of nucleolin. J Cell Sci.

[B70] Srivastava M, Pollard HB (1999). Molecular dissection of nucleolin's role in growth and cell proliferation: new insights. FASEB J.

[B71] Shibata Y, Muramatsu T, Hirai M, Inui T, Kimura T, Saito H, McCormick LM, Bu G, Kadomatsu K (2002). Nuclear targeting by the growth factor midkine. Mol Cell Biol.

[B72] Joo EJ, ten Dam GB, van Kuppevelt TH, Toida T, Linhardt RJ, Kim YS (2005). Nucleolin: acharan sulfate-binding protein on the surface of cancer cells. Glycobiology.

[B73] Callebaut C, Nisole S, Briand JP, Krust B, Hovanessian AG (2001). Inhibition of HIV infection by the cytokine midkine. Virology.

[B74] Said EA, Krust B, Nisole S, Svab J, Briand JP, Hovanessian AG (2002). The anti-HIV cytokine midkine binds the cell surface-expressed nucleolin as a low affinity receptor. J Biol Chem.

[B75] Christian S, Pilch J, Akerman ME, Porkka K, Laakkonen P, Ruoslahti E (2003). Nucleolin expressed at the cell surface is a marker of endothelial cells in angiogenic blood vessels. J Cell Biol.

[B76] Legrand D, Vigie K, Said EA, Elass E, Masson M, Slomianny MC, Carpentier M, Briand JP, Mazurier J, Hovanessian AG (2004). Surface nucleolin participates in both the binding and endocytosis of lactoferrin in target cells. Eur J Biochem.

[B77] Laber DA, Choudry MA, Taft BS, Bhupalam L, Sharma VR, Hendler FJ, Barnhart KM (2004). A phase I study of AGRO100 in advanced cancer. Journal of Clinical Oncology.

[B78] Barnhart KM, Laber DA, Bates PJ, Trent JO, Miller DM (2004). AGRO100: The translation from lab to clinic of a tumor-targeted nucleic acid aptamer. Journal of Clinical Oncology.

[B79] Ozen M, Multani AS, Chang S-M, Von Eschenbach AC, Chung LWK, Pathak S (1996). Establishment of an in vitro cell model system to study human prostate carcinogenesis: involvement of chromosome 5 in early stages of neoplastic transformation. Internation Journal of Oncology.

[B80] Kleinman HK, McGarvey ML, Hassell JR, Martin GR (1983). Formation of a supramolecular complex is involved in the reconstitution of basement membrane components. Biochemistry.

[B81] Davis LA, Ogle RC, Little CD (1989). Embryonic heart mesenchymal cell migration in laminin. Dev Biol (N Y 1985).

[B82] Vafa A, Zhang Y, Sikes RA, Marengo SR (1998). Overexpression of p185erbB2/neu in the NbE prostatic epithelial cell line increases cellular spreading and the expression of integrin alpha6beta1. Int J Oncol.

[B83] Carmichael J, DeGraff WG, Gazdar AF, Minna JD, Mitchell JB (1987). Evaluation of a tetrazolium-based semiautomated colorimetric assay: assessment of radiosensitivity. Cancer Res.

[B84] Romijn JC, Verkoelen CF, Schroeder FH (1988). Application of the MTT assay to human prostate cancer cell lines in vitro: establishment of test conditions and assessment of hormone-stimulated growth and drug-induced cytostatic and cytotoxic effects. Prostate.

[B85] Smart EJ, Ying YS, Mineo C, Anderson RG (1995). A detergent-free method for purifying caveolae membrane from tissue culture cells. Proc Natl Acad Sci U S A.

[B86] Wu HC, Hsieh JT, Gleave ME, Brown NM, Pathak S, Chung LW (1994). Derivation of androgen-independent human LNCaP prostatic cancer cell sublines: role of bone stromal cells. Int J Cancer.

[B87] Yamada KM, Even-Ram S (2002). Integrin regulation of growth factor receptors. Nat Cell Biol.

[B88] You X, Yu HM, Cohen-Gould L, Cao B, Symons M, Vande Woude GF, Knudsen BS (2003). Regulation of migration of primary prostate epithelial cells by secreted factors from prostate stromal cells. Exp Cell Res.

[B89] Comoglio PM (2001). Pathway specificity for Met signalling. Nat Cell Biol.

[B90] Zarnegar R, DeFrances MC, Oliver L, Michalopoulos G (1990). Identification and partial characterization of receptor binding sites for HGF on rat hepatocytes. Biochem Biophys Res Commun.

[B91] Wielenga VJ, van der Voort R, Taher TE, Smit L, Beuling EA, van Krimpen C, Spaargaren M, Pals ST (2000). Expression of c-Met and heparan-sulfate proteoglycan forms of CD44 in colorectal cancer. Am J Pathol.

[B92] van der Voort R, Taher TE, Wielenga VJ, Spaargaren M, Prevo R, Smit L, David G, Hartmann G, Gherardi E, Pals ST (1999). Heparan sulfate-modified CD44 promotes hepatocyte growth factor/scatter factor-induced signal transduction through the receptor tyrosine kinase c-Met. J Biol Chem.

[B93] Pollack AL, Apodaca G, Mostov KE (2004). Hepatocyte growth factor induces MDCK cell morphogenesis without causing loss of tight junction functional integrity. Am J Physiol Cell Physiol.

[B94] Derksen PW, Keehnen RM, Evers LM, van Oers MH, Spaargaren M, Pals ST (2002). Cell surface proteoglycan syndecan-1 mediates hepatocyte growth factor binding and promotes Met signaling in multiple myeloma. Blood.

[B95] Matsumoto K, Nakamura T (2001). Hepatocyte growth factor: renotropic role and potential therapeutics for renal diseases. Kidney Int.

[B96] Orrick LR, Olson MO, Busch H (1973). Comparison of nucleolar proteins of normal rat liver and Novikoff hepatoma ascites cells by two-dimensional polyacrylamide gel electrophoresis. Proc Natl Acad Sci U S A.

[B97] Derenzini M, Sirri V, Trere D, Ochs RL (1995). The quantity of nucleolar proteins nucleolin and protein B23 is related to cell doubling time in human cancer cells. Lab Invest.

[B98] Tawfic S, Goueli SA, Olson MO, Ahmed K (1994). Androgenic regulation of phosphorylation and stability of nucleolar protein nucleolin in rat ventral prostate. Prostate.

[B99] Xu LL, Su YP, Labiche R, Segawa T, Shanmugam N, McLeod DG, Moul JW, Srivastava S (2001). Quantitative expression profile of androgen-regulated genes in prostate cancer cells and identification of prostate-specific genes. Int J Cancer.

[B100] Mi Y, Thomas SD, Xu X, Casson LK, Miller DM, Bates PJ (2003). Apoptosis in leukemia cells is accompanied by alterations in the levels and localization of nucleolin. J Biol Chem.

[B101] Bates PJ, Kahlon JB, Thomas SD, Trent JO, Miller DM (1999). Antiproliferative activity of G-rich oligonucleotides correlates with protein binding. J Biol Chem.

[B102] Sikes RA, Cooper CR, Beck GL, Pruitt F, Brown ML, Balian G (2005). Bone stromal cells as therapeutics targets in osseous metastasis.

[B103] Bugler B, Caizergues-Ferrer M, Bouche G, Bourbon H, Amalric F (1982). Detection and localization of a class of proteins immunologically related to a 100-kDa nucleolar protein. Eur J Biochem.

[B104] Hovanessian AG, Puvion-Dutilleul F, Nisole S, Svab J, Perret E, Deng JS, Krust B (2000). The cell-surface-expressed nucleolin is associated with the actin cytoskeleton. Exp Cell Res.

[B105] Pfeifle J, Anderer FA (1983). Isolation and characterization of phosphoprotein pp 105 from simian virus 40-transformed mouse fibroblasts. Biochim Biophys Acta.

[B106] Pfeifle J, Hagmann W, Anderer FA (1981). Cell adhesion-dependent differences in endogenous protein phosphorylation on the surface of various cell lines. Biochim Biophys Acta.

[B107] Borer RA, Lehner CF, Eppenberger HM, Nigg EA (1989). Major nucleolar proteins shuttle between nucleus and cytoplasm. Cell.

[B108] Larrucea S, Gonzalez-Rubio C, Cambronero R, Ballou B, Bonay P, Lopez-Granados E, Bouvet P, Fontan G, Fresno M, Lopez-Trascasa M (1998). Cellular adhesion mediated by factor J, a complement inhibitor. Evidence for nucleolin involvement. J Biol Chem.

[B109] Becker J, Craig EA (1994). Heat-shock proteins as molecular chaperones. Eur J Biochem.

[B110] Lee CH, Chang SC, Chen CJ, Chang MF (1998). The nucleolin binding activity of hepatitis delta antigen is associated with nucleolus targeting. J Biol Chem.

[B111] Take M, Tsutsui J, Obama H, Ozawa M, Nakayama T, Maruyama I, Arima T, Muramatsu T (1994). Identification of nucleolin as a binding protein for midkine (MK) and heparin-binding growth associated molecule (HB-GAM). J Biochem (Tokyo).

[B112] Kleinman HK, Weeks BS, Cannon FB, Sweeney TM, Sephel GC, Clement B, Zain M, Olson MO, Jucker M, Burrous BA (1991). Identification of a 110-kDa nonintegrin cell surface laminin-binding protein which recognizes an A chain neurite-promoting peptide. Arch Biochem Biophys.

[B113] Kibbey MC, Johnson B, Petryshyn R, Jucker M, Kleinman HK (1995). A 110-kD nuclear shuttling protein, nucleolin, binds to the neurite-promoting IKVAV site of laminin-1. J Neurosci Res.

[B114] Yu D, Schwartz MZ, Petryshyn R (1998). Effect of laminin on the nuclear localization of nucleolin in rat intestinal epithelial IEC-6 cells. Biochem Biophys Res Commun.

[B115] Lozano E, Betson M, Braga VM (2003). Tumor progression: Small GTPases and loss of cell-cell adhesion. Bioessays.

[B116] Titus B, Schwartz MA, Theodorescu D (2005). Rho proteins in cell migration and metastasis. Crit Rev Eukaryot Gene Expr.

[B117] Gomez del Pulgar T, Benitah SA, Valeron PF, Espina C, Lacal JC (2005). Rho GTPase expression in tumourigenesis: evidence for a significant link. Bioessays.

[B118] Schwartz M (2004). Rho signalling at a glance. J Cell Sci.

[B119] Ridley AJ (1995). Intracellular regulation. Rac and Bcr regulate phagocytic phoxes. Curr Biol.

